# Competing risk nomogram predicting initial loco-regional recurrence in gastric cancer patients after D2 gastrectomy

**DOI:** 10.1186/s13014-019-1332-y

**Published:** 2019-07-17

**Authors:** Shu-Bei Wang, Wei-Xiang Qi, Jia-Yi Chen, Cheng Xu, Youlia M. Kirova, Wei-Guo Cao, Rong Cai, Lu Cao, Min Yan, Gang Cai

**Affiliations:** 1 Department of Radiation Oncology, Ruijin Hospital, Shanghai Jiaotong University School of Medicine, No. 197 Rui Jin Er Road, Shanghai, 200025 China; 2 Department of Radiation Oncology, Institute Curie, Paris, France; 3 Department of General Surgery, Ruijin Hospital, Shanghai Jiaotong University School of Medicine, Shanghai, China

**Keywords:** Gastric carcinoma, Loco-regional recurrence, Para-aortic lymph nodes, Radiation target volume, Competing risk nomogram

## Abstract

**Background:**

Lacking quantitative evaluations of clinicopathological features and the risk factors for loco-regional recurrence (LRR) in gastric cancer after D2 gastrectomy, we aimed to develop a competing risk nomogram to identify the risk predictors for initial LRR.

**Methods:**

We retrospectively analysed 1105 patients who underwent radical gastrectomy with D2 resection for stage I-III gastric cancer. A nomogram predicting initial LRR of gastric cancer was conducted based on Fine and Grey’s competing risk analysis. The predictive accuracy and discriminative ability of the model were determined using the concordance index (C-index) and calibration curve. Decision tree analysis was performed for patient grouping.

**Results:**

At a median follow-up of 28.4 months, 274 patients developed 373 first recurrence events (local, regional, and distant disease). The median recurrence-free survival (RFS) was 16.7 months. Multivariate competing risk analysis showed that age (SHR, 1.72; 95% CI, 1.10–2.83, *p* = 0.031), CEA (SHR, 1.94; 95% CI, 1.09–3.46, *p* = 0.024), pT4 (SHR, 2.77; 95% CI, 1.01–7.57, *p* = 0.047), lymph node metastasis (SHR 1.92, 95% CI: 1.09–3.38, *p* = 0.024) and LVI (SHR, 1.84; 95% CI, 1.06–3.20, *p* = 0.028) were independent risk factors for LRR (all *p* < 0.05). The nomogram incorporating these factors achieved good agreement between prediction and actual observation with a concordance index of 0.738 (95% CI, 0.767 to 0.709). In a subgroup analysis of node-positive patients, pN3b was associated with increased peritoneal and distant metastasis (*p* = 0.048). The para-aortic lymph nodes were the most frequent sites (*n* = 71) of LRR, and among them, the 16a2 and 16b1 nodes exhibited even more prevalence (90.1 and 81.7%).

**Conclusions:**

Adjuvant radiotherapy might be recommended in gastric cancer patients ≥65 years old or those with pN+, pT4, LVI, or increased CEA levels, particularly in high-risk or pN1-3a patients. The competing risk nomograms may be considered as convenient and individualized predictive tools for LRR in gastric cancer after D2 gastrectomy. It is also recommended that the clinical target volume (CTV) include 16a2 and 16b1 regions of para-aortic lymph nodes.

## Background

Globally, there are nearly 1 million cases of gastric cancer annually, making it the third leading cause of global cancer mortality [1]. China is one of the countries with a high gastric cancer incidence [2]. Most patients with gastric cancer in China are symptomatic and are diagnosed with locally advanced disease [3]. Complete surgical eradication of a gastric tumour (total or partial gastrectomy) with resection of adjacent lymph nodes (D2 dissection) represents the best chance for long-term survival [4]. The observed high mortality reflects the prevalence of advanced disease at presentation [3]. Treatment failure in patients with resected gastric carcinoma is related to locoregional failure (LRR) as well as to distant metastases with or without peritoneal dissemination. LRR following curative resection with lymph node D0–1 resection is fairly common [5, 6]. LRR is more frequent in patients who underwent surgery without chemoradiotherapy or in those who had a low number of resected negative lymph nodes [7, 8]. Current trials of postoperative chemoradiotherapy have shown a significant survival benefit after complete resection with D0–1 resection of gastric cancer [9, 10]. However, in the Dutch CRITICS trial, 788 patients with potentially resectable gastric cancer received induction chemotherapy followed by surgery and randomization to postoperative chemotherapy or chemoradiotherapy [11]. All patients underwent D1 or better lymphadenectomy with at least 15 nodes in the resected specimens. At a median follow-up of 61 months, there were no significant differences in five-year overall survival or progression-free survival, and local recurrence rates were 15% versus 11%. Thus, selecting patients who may benefit from radiotherapy seems to be more important.

Many predicting factors associated with LRR have been identified in previous studies, such as tumour location [12], pT stage [7, 13, 14], pathologic node status [7, 13, 14], extent of lymph node dissection [12, 15], tumour grade [13], and surgical margin status [15]. Considering these factors, postoperative adjuvant therapies and radiotherapy may be individualized to decrease recurrence rates, which will help to select suitable patients for radiotherapy in a multidisciplinary approach. A nomogram is a statistical model that combines and quantifies all proven prognostic factors using a simple graphical representation. Several nomograms for gastric cancer recurrence and prognosis have been established in recent years, but none specifically for this patient population based on a competing risk model. Therefore, we conducted this retrospective study to quantitatively evaluate the clinicopathological features and the risk factors for initial LRR in gastric cancer after D2 gastrectomy by establishing a nomogram and decision trees. Our study aimed to identify the patients with the highest risk for LRR. Furthermore, the second aim was to determine the pattern and risk factors for para-aortic node recurrence.

## Methods

### Patient characteristics

Between January 2010 and December 2012, 1250 patients with adenocarcinoma of the stomach or gastroesophageal junction underwent gastric resection at our hospital.

The inclusion criteria included patients diagnosed with stage I-III adenocarcinoma according to the American Joint Committee on Cancer (AJCC) eighth edition [16]; who underwent R0 gastrectomy with D2 lymph node dissection; and with histopathologically proven adenocarcinoma of the stomach or gastroesophageal junction.

The exclusion criteria were patients who did not undergo R0 resection (*n* = 138); and patients for whom not all of the nomogram variables were available (*n* = 7).

R0 resection was defined as a total or partial gastrectomy without microscopic involvement of the resection lines. Among the remaining 1105 patients, 288 patients underwent total gastrectomy, and 817 patients had partial gastrectomy. Only 10 patients received pancreatic tail resection or splenectomy or both.

### Data collection

All patients’ clinical and pathological characteristics, including age, sex, tumour location, depth of tumour invasion, number of positive lymph nodes, neo-adjuvant treatment, adjuvant treatment, recurrence and survival information, were retrospectively reviewed based on operative notes and medical records.

### Definition of the recurrence pattern and survival time

All patients were followed from the date of surgery to the date of death or loss to follow-up. The last follow-up for recurrence was January 2018. Patients were followed up every 3 months during the first 2 years and every 6 months thereafter following surgery. Regular follow-up evaluations consisted of endoscopy, ultrasound (US), computed tomography (CT), magnetic resonance imaging (MRI) or PET/CT scans. The recurrence pattern was determined according to the primary recurrence site diagnosed by imaging (CT, US, MRI or PET/CT), endoscopy, ascitic cytology, or biopsy. All of the recurrences were evidenced by diagnostic imaging, such as endoscopy, CT, MRI or PET/CT. Furthermore, histological examinations were performed in 137 patients. Local regional recurrence included recurrence at stomas, duodenal stump, tumour bed, residual stomach and regional lymph nodes. The regions of lymph node recurrence were determined according to the Japanese Gastric Cancer Treatment Guidelines [17]. Peritoneal dissemination was determined to be any recurrence within the abdominal cavity due to intraperitoneal distribution or mesothelial implantation. Distant metastasis was defined as any metastasis to distant lymph nodes outside the abdominal cavity, distant organs or sites except for the peritoneum [18]. Recurrence-free survival (RFS) was defined as the time from surgery to recurrence or death from any other cause. The death of patients who did not present to the hospital was confirmed through phone conversations or letters.

In the Japanese Gastric Cancer Association (JGCA) N-classification, every single lymph node was numbered as a station (No. 1 to No. 112) and grouped by anatomical position. According to the Japanese classification, the para-aortic lymph node is further numbered as station 16 (No. 16) and sub-classified as 16a1, 16a2, 16b1 and 16b2 [17]. The ‘16a1’ lymph node is defined as a node located between the aortic foramen and the upper margin of the celiac axis in the craniocaudal direction. The ‘16a2’ lymph node is defined as a node located between the upper margin of the celiac axis and the lower margin of the left renal vein (LRV). The ‘16b1’ lymph node is defined as a node located between the lower margin of LRV and the upper margin of the inferior mesenteric artery (IMA). The ‘16b2’ lymph node is defined as a node located between the upper margin of the IMA and bifurcation of the abdominal aorta [17].

### Statistical analysis

The primary end point of this study was LRR and its risk factors. The secondary end point was the pattern and risk factors for para-aortic node recurrence.

Statistical analyses were performed using SPSS Statistics 19 (IBM Corp., Armonk, NY, USA). Thirteen clinically relative characteristics (sex, age, Bormann type, tumour location, histological type, CEA level, CA-199 level, T stage, lymph node metastasis, lymphovascular invasion, perineural invasion, adjuvant chemotherapy, and number of lymph nodes dissected) were examined as potential risk factors for recurrence. Kaplan-Meier analysis and log-rank tests were used to assess associated factors. Initial local regional recurrence and initial distant metastasis with or without LRR were regarded as two competing events. The combined effects of the variables on LRR were evaluated by proportional hazard analyses of Fine and Grey’s model [19, 20]. Predictors with a *P* value of < 0.05 in the univariate analysis were entered into a multivariate analysis based on proportional subdistribution hazard models, and a nomogram was developed based on the independent risk factors identified in the multivariate analysis. Nomograms were created using the nomogram function of the “rms” package in R software, and the performance of the nomograms was measured by the concordance index (C-index) and assessed by calibration curves [21]. A higher C-index indicates a better ability to separate patients with different survival outcomes. The calibration curves were used to compare the predicted probability with the observed probability in the study cohort. Bootstrapping with 100 resamples was used to develop the nomogram and calibration curve to reduce the overfit bias [22]. Competing risk analysis, nomogram and ROC curves were determined using R version 3.4.2 software (The R Foundation for Statistical Computing, Vienna, Austria. http://www.r-project.org). A two-tailed *p*-value < 0.05 was considered statistically significant. The program partykit was implemented in R software (Mathsoft, Cambridge, MA) was used to generate a decision tree to validate the nomogram.

## Results

The clinical characteristics of the 1105 patients are summarized in Table 1. The median age at diagnosis was 59 years (range, 22–88). There were 785 males (71.0%) and 320 females (29.0%). The most frequent tumour location was the stomach antrum (46.4%) and body (39.5%), followed by fundus (14.1%). The median number of dissected lymph nodes was 18 (15–81). Neoadjuvant and adjuvant chemotherapy were administered in 23 (2.1%) and 681 (61.6%) patients, respectively. A total of 677 patients (61.3%) received fluorouracil-based chemotherapy. Almost half of the patients (48.4%) received adjuvant chemotherapy over 3 cycles. A total of 144 patients received oral administration of S-1 or capecitabine for approximately 1 year or until the side effects became too strong to tolerate. No patient received adjuvant radiotherapy.

**Table 1:** Clinicopathological characteristics of 1105 gastric cancer patients after D2 gastrectomy

Clinical features	No. of patients (*n* = 1105)
Sex, n (%)	
Male	785 (71.0%)
Female	320 (29.0%)
Age, median year (range)	59 (22~88)
Bormann type, n (%)	
I	136 (12.3%)
II	425 (38.5%)
III	506 (45.8%)
IV	38 (3.4%)
Tumor location, n (%)	
Upper one-third	156 (14.1%)
Middle one-third	436 (39.5%)
Lower one-third	513 (46.4%)
Type of resection, n (%)	
total gastrectomy	288 (26.1%)
subtotal / partial gastrectomy	817 (73.9%)
Histological differentiation, n (%)	
Well-moderate differentiated tumors	242 (21.9%)
Poorly differentiated and undifferentiated tumors	836 (78.1%)
T stage, n (%)	
pT1	283 (25.6%)
pT2	187 (16.9%)
pT3	251 (22.7%)
pT4	384 (34.8%)
N stage, n (%)	
pN0	469 (42.4%)
pN1	197 (17.8%)
pN2	207 (18.7%)
pN3a	165 (14.9%)
pN3b	67 (6.1%)
AJCC stage, n (%)	
IA	227 (20.5%)
IB	129 (11.7%)
IIA	133 (12.0%)
IIB	156 (14.1%)
IIIA	243 (22.0%)
IIIB	151 (13.7%)
IIIC	66 (6.0%)
LVI	
Positive	206 (18.6%)
Negative	899 (81.4%)
Perineural invasion	
Positive	248 (22.4%)
Negative	857 (77.6%)
Neo-adjuvant chemotherapy	
Yes	23 (2.1%)
No	1082 (97.9%)
Adjuvant chemotherapy	
Yes	681 (61.6%)
No	424 (38.4%)

### Initial patterns of failure in patients with gastric cancer after radical gastrectomy

**Fig. 1:**
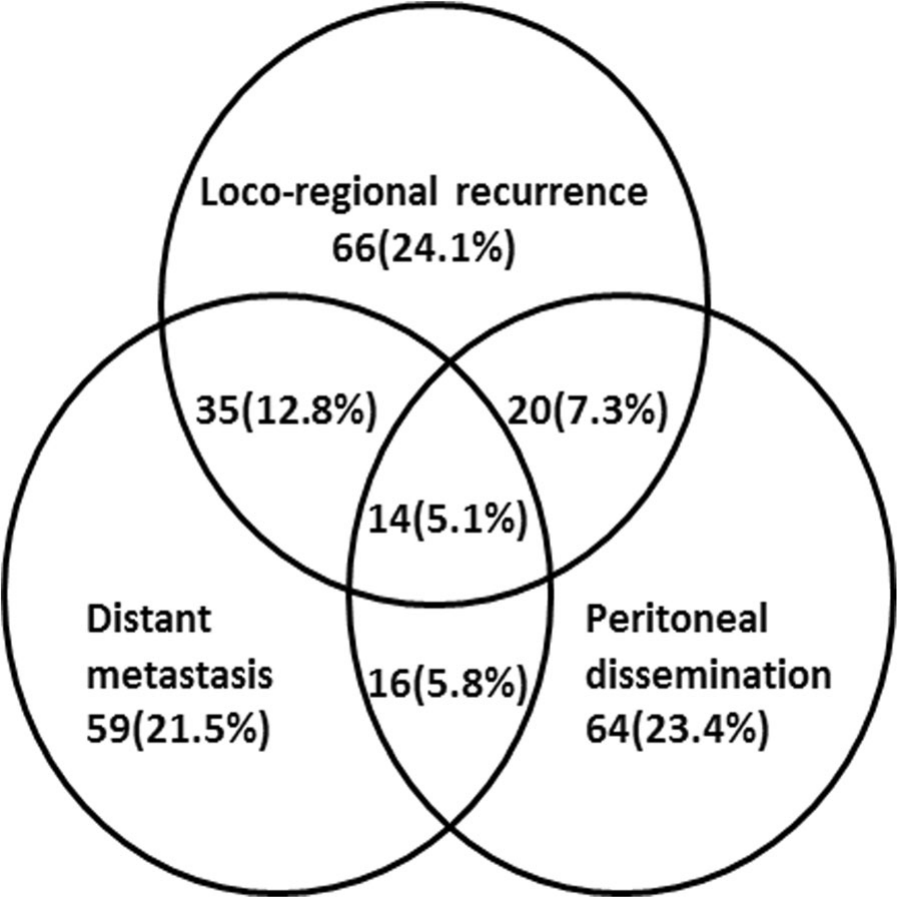
Initial recurrence patterns of gastric cancer patients undergoing D2 gastrectomy

With a median follow-up of 28.4 (range, 3–94) months, 274 patients (24.8%) of 1105 patients developed recurrences. In total, there were 373 first recurrence events, including LRR (*n* = 135), distant metastasis (*n* = 124) and peritoneal dissemination (*n* = 114). Eighty-five patients experienced multiple patterns of first failure. Among the patients who experienced recurrence at a single site, the numbers of patients with LRR, distant metastasis and peritoneal dissemination were 66, 59 and 64, respectively (Fig. 1). The most common sites of LRR included regional node recurrence (*n* = 95).

### Risk factors for LRR

By univariate competing risk regression analysis, age of 65 years or older, gastric body tumour location, pN+, pN3, pT4, lymphovascular invasion (LVI), perineural invasion, and elevated CEA and CA-199 levels were correlated with LRR (Table 2, all *p* < 0.05).

**Table 2:** Univariate and multivariate competing risk analysis of factors associated with risk of loco-regional recurrence

Factors	n	Univariate		Multivariate	
		SHR.95%CI	*p*-value	SHR,95%CI	*p*-value
Sex					
Male	791	Reference		–	–
Female	321	0.59 (0.32–1.08)	0.085	–	–
Age (years)					
<65	743	Reference		Reference	
≥ 65	369	2.02 (1.24–3.27)	0.004	1.72 (1.10–2.83)	0.031
Bormann type					
I-III	1065	Reference		–	–
IV	38	1.32 (0.93–1.88)	0.12	–	–
Tumor location					
Upper one-third	156	Reference		Reference	
Middle one-third	436	0.34 (0.16–0.72)	0.005	0.42 (0.20–0.90)	0.026
Lower one-third	513	0.78 (0.42–1.48)	0.46	0.94 (0.49–1.77)	0.84
Histological type					
Well-moderate differentiated	235	Reference		–	–
Poorly differentiated and undifferentiated	840	1.53 (0.78–2.99)	0.21	–	–
CEA					
Normal	839	Reference		Reference	
Increase	191	2.62 (1.57–4.37)	< 0.001	1.94 (1.09–3.46)	0.024
CA-199					
Normal	921	Reference		Reference	
Increase	112	2.08 (1.12–3.89)	0.021	1.04 (0.50–2.18)	0.92
T stage					
pT1	283	Reference		Reference	
pT2	178	3.72 (1.33–10.44)	0.012	2.64 (0.93–7.54)	0.07
pT3	251	2.61 (0.91–7.44)	0.074	1.48 (0.50–4.35)	0.47
pT4	393	5.89 (2.35–14.74)	< 0.001	2.77 (1.01–7.57)	0.047
Lymph node metastasis					
No	435	Reference		Reference	
Yes	619	2.88 (1.57–5.30)	0.001	1.92 (1.09–3.38)	0.024
LVI					
Negative	851	Reference		Reference	
Positive	206	2.48 (1.48–4.14)	0.001	1.84 (1.06–3.20)	0.028
Perineural invasion					
Negative	807	Reference		Reference	
Positive	250	1.64 (0.98–2.77)	0.062	1.11 (0.65–1.91)	0.71
Adjuvant chemotherapy					
No	427	Reference		–	–
Yes	684	1.23 (0.73–2.08)	0.43	–	–
No. of lymph node dissection					
< 25	847	Reference		–	–
≥ 25	258	1.24 (0.72–2.13)	0.44	–	–

*Abbreviations*: *CI* Confidence interval, *SHR* Subdistribution hazard regression

**Fig. 2:**
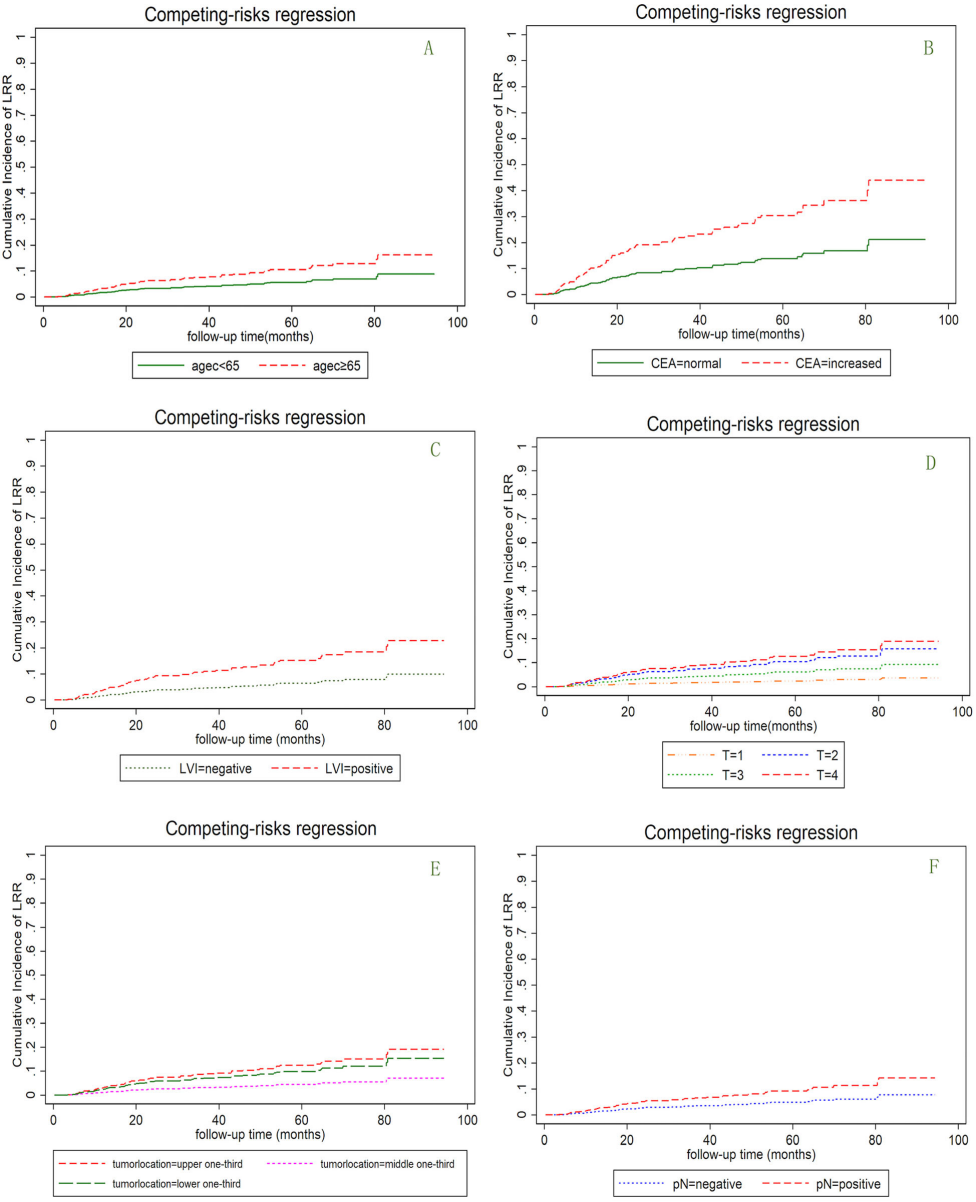
**a**: Comparison of cumulative incidence of LRR between the patients <65 years old and ≥ 65 years old. LRR = local regional recurrence. **b**: Comparison of cumulative incidence of LRR between the patients with normal and increased CEA level. **c**: Comparison of cumulative incidence of LRR between the patients with negative and positive LVI. **d**: Comparison of cumulative incidence of LRR among the patients with different T stages. **e**: Comparison of cumulative incidence of LRR among the patients with different tumor locations. **f**: Comparison of cumulative incidence of LRR between the node-negative and node-positive patients

Using the multivariate competing risk regression analysis, we found age of 65 years or older (SHR = 1.72, 95% CI 1.10–2.83, *p* = 0.031), pN+ (SHR = 1.92, 95% CI 1.09–3.38, *p* = 0.024), pT4 (SHR = 2.77, 95% CI 1.01–7.57, *p* = 0.047), LVI (SHR = 1.84, 95% CI 1.06–3.20, *p* = 0.028) and elevated CEA level (SHR = 1.94, 95% CI 1.09–3.46, *p* = 0.024) to be significantly associated with LRR (Fig. 2).

### Construction and validation of nomograms for initial LRR

**Fig. 3:**
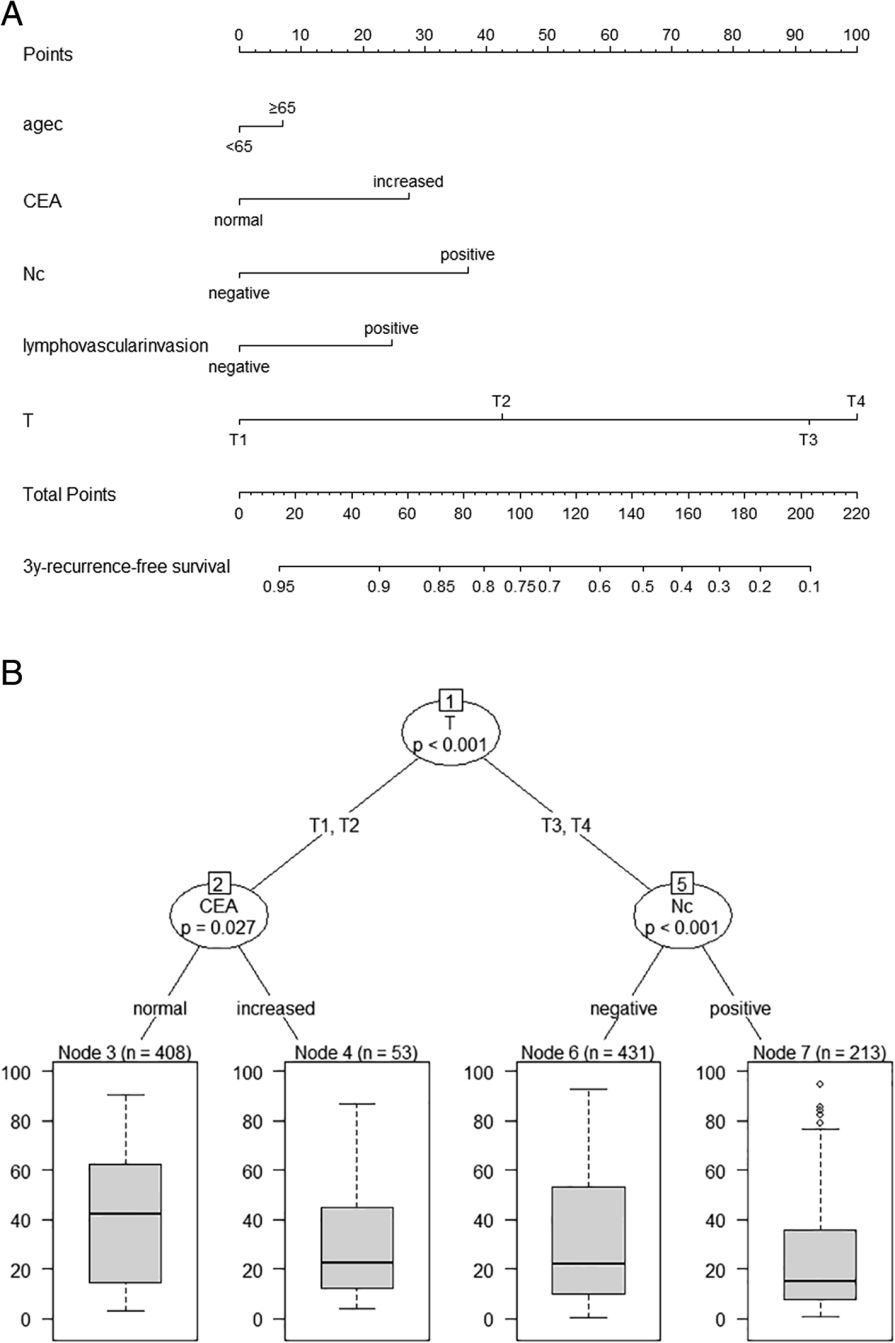
**a**: Nomograms for predicting RFS of gastric cancer after D2 gastrectomy. Each variable corresponds to a point on the scale. According to the sum of these points projected on the bottom scales, the nomogram can provide the probabilities of 3-year RFS for an individual patient. RFS = recurrence-free survival. **b**: Best tree for recurrence-free survival of gastric cancer. Pathological T stage was the initial node, preoperative CEA levels and N stage following D2 gastrectomy

All independent predictors of initial LRR in the entire study cohort were integrated into the nomogram. Figure 3a illustrates the predictive nomograms that were established for the 3-year RFS rate. A patient’s individual probability of survival can easily be calculated by adding the scores for each selected variable. The nomogram demonstrated good accuracy for RFS prediction, with a C-index of 0.738 (95% CI, 0.767 to 0.709). Calibration plots for the probabilities of 3-year RFS showed fair agreement between the nomogram-predicted survival and actual survival (Fig. 3b).

### Classification tree for the prognosis of gastric cancer

**Fig. 4:**
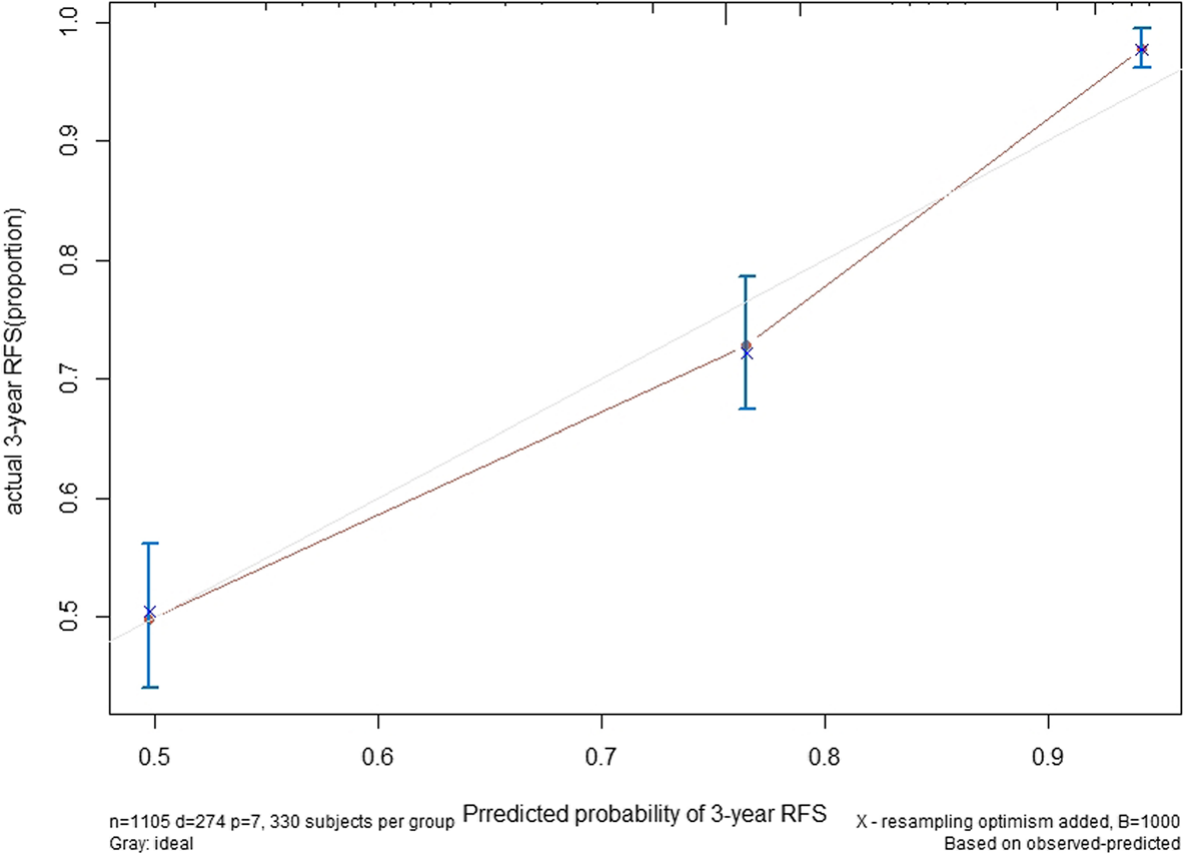
Calibration plots for recurrence-free survival in patients with gastric cancer. The solid grey line represents equality between the predicted and observed probabilities. With the red line close to the solid grey line, the plots reveal excellent agreement between the nomogram-predicted probabilities and actual observations

Classification tree analysis is another method for determining the recurrence factors associated with gastric cancer after D2 gastrectomy. The result of the pruned tree is shown in Fig. 4. The pathological T stage was the initial node. Preoperative CEA level and pathological N stage were also determinants for RFS in this patient population.

### Estimation of the recurrence period

During the follow-up, 274 patients experienced recurrence with a median RFS of 16.7 months (interquartile range [IQR] 8.5, 30.8). The median RFS rates for patients with LRR, distant metastasis, peritoneal dissemination and multiple pattern recurrence as the first event were 18.1 months (IQR 10.6, 39.2), 15.8 months (IQR 7.8, 30.7), 14.3 months (IQR 8.4, 22.7), and 17.3 months (IQR 7.8, 25.8), respectively (*p* = 0.178).

We then grouped patients according to risk factors (pN+, pT4, LVI, ≥65 years old or elevated CEA) for LRR: the high-risk group had 4–5 risk factors (LRR rate 30.1%); the intermediate-risk group had 2–3 risk factors (LRR rate 17.8%); and the low-risk group had 0–1 risk factor (LRR rate 4.7%) (*p* < 0.0001).

**Fig. 5:**
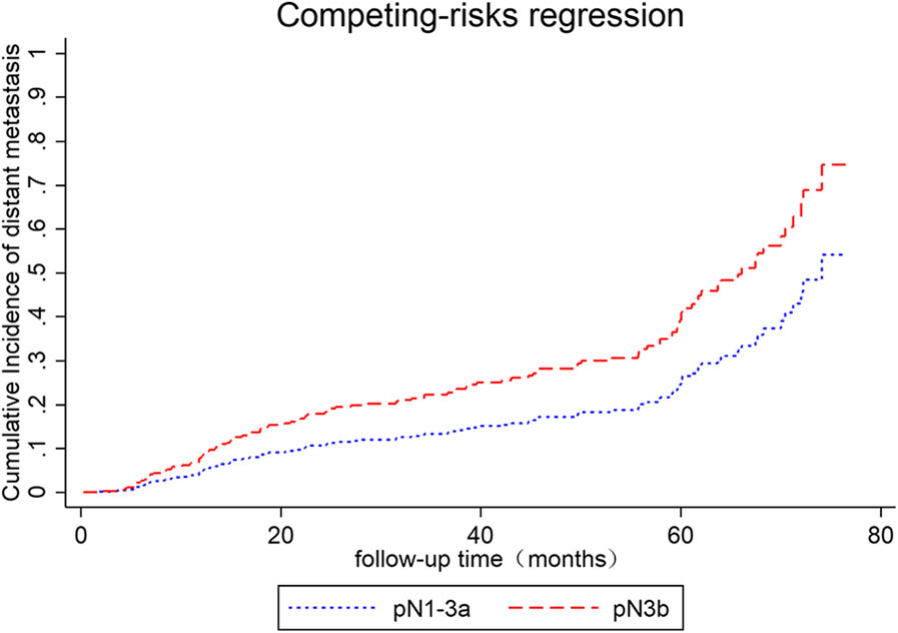
Cumulative incidence of distant metastasis stratified by pN stage in node-positive gastric cancer patients

Finally, we analysed the subgroup of node-positive patients. Using multivariate competing risk regression analysis, the risk of developing distant metastasis in patients with pN3b gastric cancer was significantly higher than that in pN1-3a gastric patients (SHR 1.68, 95% CI: 1.00–2.83, *p* = 0.048, Fig. 5).

### Pattern and risk factors for Para-aortic node recurrence

The para-aortic lymph node was the most frequent site of regional node recurrence (71 patients, 74.7%). Kaplan-Meier analysis was conducted to investigate the prognostic factors in patients with para-aortic lymph node recurrence (Table 3). LVI, pN+, pT3/4, pN3 and elevated CEA or CA-199 levels were significantly associated with para-aortic lymph node recurrence in a univariate analysis (*p* < 0.05). However, there was no significant difference in the para-aortic lymph node metastasis rate among different tumour locations. In the multivariate analysis, pN3 (HR = 2.737, 95% CI 1.430–5.240, *p* = 0.002), LVI (HR = 2.399, 95% CI 1.354–4.248, *p* = 0.003) and elevated CEA (HR = 4.598, 95% CI 2.570–8.226, *p* < 0.001) showed a statistically significant correction with para-aortic lymph node recurrence. The sites of para-aortic node recurrence were 16a2 (*n* = 64/71, 90.1%), 16b1 (*n* = 58/71, 81.7%), 16b2 (*n* = 26/71, 36.6%) and 16a1 (*n* = 21/71, 29.6%).

**Table 3:** Univariate and multivariate analysis of para-aortic lymph node recurrence

Factors	n	Univariate analysis		Multivariate analysis	
		3-year RFS (%)	*p*-value	HR (95%CI)	*p*-value
CEA			< 0.001		< 0.001
Increase	191	78.7		4.60 (2.57–8.23)	
Normal	839	95.6		1	
CA-199			0.007		0.510
Increase	112	87.1		0.77 (0.36–1.67)	
Normal	921	93.4		1	
T stage			< 0.001		0.29
T3–4	628	88.7		1.53 (0.69–3.39)	
T1–2	449	94.3		1	
pN+			< 0.001		0.125
Yes	619	88.3		2.17 (0.81–5.82)	
No	435	98.0		1	
N stage			< 0.001		0.002
pN3	232	78.5		2.74 (1.43–5.24)	
pN0–2	822	95.9		1	
LVI			< 0.001		0.003
Positive	206	81.5		2.40 (1.35–4.25)	
Negative	851	95.3		1	
Tumor location			0.722	–	–
Upper one-third	156	93.8		–	–
Middle one-third	436	93.8		–	–
Lower one-third	513	91.8		–	–

## Discussion

In patients with localized or locally advanced gastric cancer, prognosis remains dismal after surgery alone. Efforts to improve treatment results beyond those obtained with surgery alone have included chemotherapy and radiotherapy. Intergroup study 0116 showed survival improvement in association with postoperative chemoradiotherapy [9, 23]. The unsatisfactory results of this study are related to the limited extent of the surgical procedure in most cases. Previously, evidence was provided by the ARTIST trial with 458 patients treated with D2 lymph node dissection [24]. LRR was significantly lower in patients who received chemoradiotherapy (7 versus 13%, *p* = 0.0033). However, survival rates were similar in both arms [24]. Chemoradiotherapy seemed to be beneficial in patients with node-positive disease or a higher lymph node ratio. The available data on whether there is a survival benefit from postoperative radiotherapy in patients receiving radical D2 gastrectomy is controversial [25, 26]. On the other hand, the use of image-guided intensity-modulated radiation therapy reduces the incidence of gastrointestinal toxicity. Therefore, the interest of the present study is to select patients who may benefit from radiotherapy and to determine a suitable radiation target volume for adjuvant radiotherapy. The results of this study show the following (1): multiple factors can be taken into account to more accurately predict the risk of LRR in patients treated with D2 gastrectomy, including age, tumour stage, LVI and CEA level; and (2) the 16a2 and 16b1 regions of para-aortic lymph nodes should be included in postoperative clinical target volume (CTV) planning in patients undergoing D2 gastrectomy.

Our study confirms that LRR is relatively common after D2 gastrectomy in patients with gastric cancer. One problem in designing prospective trials of adjuvant RT is selecting patients who might have the highest chance to benefit from local control. Studies have identified many factors associated with loco-regional failure in univariate analysis, including tumour location [12], pT stage [7, 13, 14], pathologic node status [7, 13, 14], tumour grade [13], surgical margin status [15], extent of lymph node dissection [12, 15], and tumour size [13].

The present study shows that multiple factors allow a better stratification of the risk of LRR for patients undergoing D2 gastrectomy. Patients with 4–5 risk factors (≥65 years old, pN+, pT4, LVI or elevated CEA level), which we define here as conferring high risk, with an LRR rate of up to 30.1%, have more necessity for radiotherapy.

In this analysis, we demonstrate the feasibility of this nomogram and its ability to predict probabilities using points on a scale from 0 to 100 via a user-friendly graphic interface with readily available clinicopathological data. The nomogram supports the use of radiotherapy in the “suitable” group, which is defined as patients with 4–5 risk factors (≥65 years old, pN+, pT4, LVI or elevated CEA level). The nomogram performs well in predicting RFS, and its prediction is supported by the C-index (0.738) and the calibration curve.

However, in the subgroup analyses of our study, although most (76.4%) of the patients with pN+ received adjuvant chemotherapy, the rate of LRR (18.1%) remained high. Nevertheless, as found in other studies [24, 27], peritoneal metastasis and distant metastasis (27.0%) were even more frequent than LRR. As shown from the analysis in the node-positive subgroup, pN3b patients suffer earlier distant metastasis or peritoneal dissemination than patients with pN1-3a. These results provide support for adjuvant chemoradiotherapy in pN1-3a patients with D2-resected gastric cancer who may benefit from local treatment.

The para-aortic lymph node is the most frequent site of regional node recurrence in patients with D2-resected gastric cancer. It has been reported that the incidence of recurrence in the para-aortic region is 61.5% in 16b nodes and 58.2% in 16a nodes in N3 patients after D2 lymph node dissection [28]. In our study, the incidence of para-aortic lymph node recurrence was high, which is similar to the findings reported in other studies [15]. The 2015 Cochrane analysis of the JCOG trial and two other smaller randomized trials of D2 versus D3 (with paraaortic lymphadenectomy) dissection concluded that resection of the paraaortic nodes did not provide any significant survival benefit [29]. Thus, the surgical treatment of para-aortic lymph node dissection (D3) is not yet established. Meanwhile, the para-aortic lymph node region is not included in the target of postoperative radiation according to the National Comprehensive Cancer Network (NCCN) guidelines [30] or the CRITICS guidelines. The CRITICS guideline recommends that CTV for adjuvant radiotherapy include the anastomoses, gastric bed/remnant and elective lymph node stations corresponding to the specific tumour location, such as the proximal, middle, and distal stomach [11]. However, because our study found that the 16a2 and 16b1 regions of the para-aortic lymph nodes were the most common sites regardless of the primary tumour location, the 16a2 and 16b1 regions are recommended for CTV inclusion. The target volume, including the 16a2 and 16b1 regions, may be larger, and radiation-induced toxicity of adjacent organs, such as the kidneys, may increase. However, great technical progress has been made to improve the effectiveness of radiotherapy and minimize the side effects [11, 24].

To our knowledge, this is the first study to apply competing risk analysis methods to risk factors for LRR in gastric carcinoma, a statistical approach that is less biased than the more commonly used Kaplan-Meier method. Our analysis of the para-aortic node recurrence subgroup identified that all patients with different tumour locations had a high probability of 16a2 and 16b1 recurrence, confirming the clinical importance of 16a2 and 16b1 region irradiation in adjuvant radiotherapy.

A limitation of our study is that this is only a retrospective study based on a single centre. Prospective studies of para-aortic region radiotherapy must be conducted in the future.

## Conclusion

The results of this analysis demonstrate that lymph node status, T stage, LVI, age and CEA level are significant factors associated with LRR for patients undergoing D2 gastrectomy. The formulation of a nomogram for calculating LRR in patients undergoing D2 gastrectomy may guide appropriate selection for patients at high risk for LRR for whom adjuvant radiotherapy would be appropriate. It is also recommended that the CTV include the 16a2 and 16b1 regions of the para-aortic lymph nodes for adjuvant radiotherapy for the treatment of gastric cancer.

## Data Availability

The datasets used and/or analysed during the current study are available from the corresponding author on reasonable request.

## Abbreviations

AJCC: The American Joint Committee on Cancer
C-Index: concordance index
CT: Computed tomography
CTV: Clinical target volume
IMA: Inferior mesenteric artery
JGCA: Japanese Gastric Cancer Association
LRR: Local regional recurrence
LRV: Left renal vein
LVI: Lymphovascular invasion
MRI: Magnetic resonance imaging
NCCN: National Comprehensive Cancer Network
RFS: Recurrence-free survival
US: Ultrasound
